# A Gateway MultiSite Recombination Cloning Toolkit

**DOI:** 10.1371/journal.pone.0024531

**Published:** 2011-09-09

**Authors:** Lena K. Petersen, R. Steven Stowers

**Affiliations:** Department of Cell Biology and Neuroscience, Montana State University, Bozeman, Montana, United States of America; Oregon Health and Science University, United States of America

## Abstract

The generation of DNA constructs is often a rate-limiting step in conducting biological experiments. Recombination cloning of single DNA fragments using the Gateway system provided an advance over traditional restriction enzyme cloning due to increases in efficiency and reliability. Here we introduce a series of entry clones and a destination vector for use in two, three, and four fragment Gateway MultiSite recombination cloning whose advantages include increased flexibility and versatility. In contrast to Gateway single-fragment cloning approaches where variations are typically incorporated into model system-specific destination vectors, our Gateway MultiSite cloning strategy incorporates variations in easily generated entry clones that are model system-independent. In particular, we present entry clones containing insertions of GAL4, QF, UAS, QUAS, eGFP, and mCherry, among others, and demonstrate their *in vivo* functionality in *Drosophila* by using them to generate expression clones including GAL4 and QF drivers for various trp ion channel family members, UAS and QUAS excitatory and inhibitory light-gated ion channels, and QUAS red and green fluorescent synaptic vesicle markers. We thus establish a starter toolkit of modular Gateway MultiSite entry clones potentially adaptable to any model system. An inventory of entry clones and destination vectors for Gateway MultiSite cloning has also been established (www.gatewaymultisite.org).

## Introduction

In the post-genomic era understanding gene function is a major goal of biological research. Studies aimed at elucidating the function of a gene often include determining its *in vivo* spatial and temporal cell type-specific expression pattern, the effects of silencing or ectopically expressing the gene, and the dynamic subcellular localization and trafficking of its encoded protein. A prerequisite for each of these analyses is usually the generation of a novel DNA construct and this is typically a laborious, time-consuming, multi-step process when using a traditional restriction enzyme-based cloning approach. The development of recombination cloning utilizing phage lambda integrase [1] (marketed as Gateway recombination cloning by Invitrogen) provided an advance in efficiency and reliability for certain cloning applications, especially those that are highly repetitive. For instance, cloning cDNAs for 1,282 Arabadopsis transcription factors into yeast expression vectors [2], cloning cDNAs for 10,167 *C.elegans* genes into *E.coli* expression vectors [3], and cloning >5000 *Drosophila* enhancers into GAL4 vectors [4].

A limitation of the original Gateway cloning technology (hereafter single-fragment Gateway cloning) is that only one DNA fragment at a time contained in an “entry” vector could be recombination cloned into a “destination” vector to create an expression clone. An advance in recombination cloning technology was the development of multi-fragment recombination cloning that allows simultaneous cloning of more than one DNA fragment into an expression vector [5] (marketed as Gateway MultiSite recombination cloning by Invitrogen). This multi-fragment recombination cloning system utilizes mutant versions of the phage lambda integrase recognition sequences that are only functional in specific combinations. The current version of Gateway MultiSite recombination cloning allows flexible, simultaneous, position and orientation-specific cloning of two, three, or four DNA fragments into a destination vector to create an expression clone.

Our intention in this work was to develop a starter toolkit of entry clones suitable for Gateway MultiSite recombination cloning. The entry clones we generated include insertions of GAL4, QF, UAS, QUAS, eGFP, and mCherry DNA fragments. We have also generated a *Drosophila* destination vector that was used to demonstrate the functionality of these and numerous other entry clones *in vivo* in *Drosophila*, but the entry clones are potentially compatible with other model systems with an appropriate destination vector. A website database of Gateway MultiSite entry clones and destination vectors has also been established (www.gatewaymultisite.org).

## Results

Similar to Gateway single-fragment recombination cloning, Gateway MultiSite recombination cloning is a two-step process. In the first step, DNA fragments (typically PCR products) containing flanking attB sites are recombination cloned via the BP reaction (a recombination reaction between attB and attP sites) into pDONR vectors containing attP sites to create “entry” clones. The entry clones are then mixed in appropriate two, three, or four fragment combinations with a “destination” vector in the LR reaction (a recombination reaction between attL and attR sites) to generate expression clones. A major difference between single-fragment Gateway cloning and Gateway MultiSite cloning is the latter utilizes six distinct pDONR vectors instead of one. These six pDONR vectors are depicted in Figures S1A-F. Each pDONR vector contains a different pair of attP sites. These distinct attP sites provide substrate specificity and are converted into distinct attL and attR sites in the BP reaction. A list of the attB sequences used for generating Gateway MultiSite entry clones via the BP reaction is shown in Table S1.

**Figure 1:**
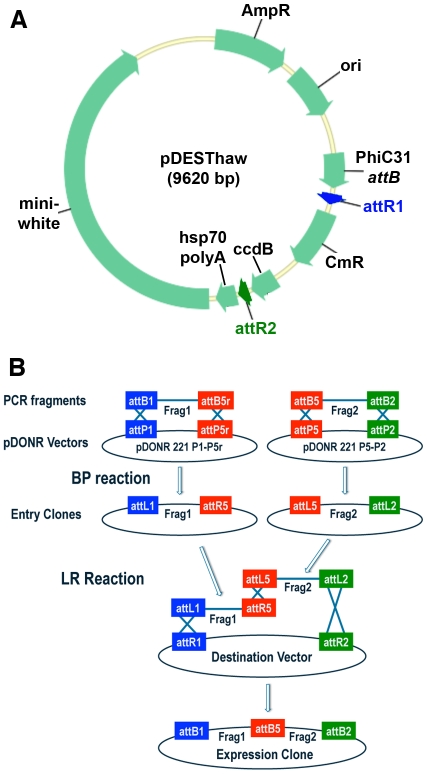
*Drosophila* destination vector and two-fragment schematic for Gateway MultiSite cloning. A) The *Drosophila* destination vector pDESThaw. This destination vector contains an hsp70 polyadenylation sequence, a *PhiC31* attB site for *PhiC31* integrase-mediate site-specific transgenesis, and mini-*white* as a transformation marker. Appropriate combinations of two, three, or four entry clones recombine in a position and orientation-specific manner with ampicillin-resistant pDESThaw in the LR reaction to generate expression clones. The LR reactions for two, three, and four fragment Gateway MultiSite recombination all use the LR Clonase II Plus enzyme mix. Both the BP and LR reactions take advantage of the Gateway cassette that includes a chloramphenicol resistance marker and the ccdB gene. The Gateway cassette is located between the attP sites in the pDONR vectors (Figure S1) and between the attR1 and attR2 sites of the pDESThaw destination vector. The ccdB gene is toxic to any bacterial strain that does not contain a genetic suppressor including most common laboratory bacterial strains used for cloning such as *DH10B* and *DH5α*. ccdB containing clones were propagated using the now discontinued bacterial strain DB3.1 that has been replaced by Invitrogen with strain ccdB Survival 2 T1R. When BP and LR reactions are transformed into non-ccdB suppressor strains, only bacteria containing clones in which the Gateway cassette has been recombined out (and presumably the fragment(s) of interest recombined in) survive on kanamycin or ampicillin-selective plates. This results in a low frequency of colonies that do not contain the insert(s) of interest. B) Schematic diagram of two-fragment Gateway MultiSite recombination cloning. Fragment 1 and fragment 2 are amplified by PCR using oligonucleotides that incorporate flanking attB1 and attB5r sites in fragment 1 and flanking attB5 and attB2 sites in fragment 2. Fragment 1 is combined with pDONR 221 P1-P5r and fragment 2 is combined with pDONR 221 P5-P2 in separate BP reactions. The products of the BP reactions are pENTR attL1-Frag1-attR5 and pENTR attL5-Frag2-attL2. In the LR reaction both of these entry clones are combined with a destination vector to produce an expression clone containing fragment 1 and fragment 2 in a position and orientation-specific manner. Note that pENTR L1-R5 entry clones are also used in four-fragment Gateway MultiSite cloning. Schematic modified from the Invitrogen MultiSite Gateway Pro user manual.

The second step of MultiSite recombination cloning involves integrating the fragments contained in the entry clones into a destination vector via the LR reaction. We decided to assess the functionality of our Gateway MultiSite entry clones in *Drosophila* and hence designed the fly destination vector pDESThaw for compatibility with two, three, or four fragment Gateway MultiSite recombination cloning. Our strategy in developing this toolkit was to incorporate into the destination vector only features common to all constructs including a polyadenylation sequence from the hsp70 gene, an attB site from phage PhiC31 for site-specific genome integration, and mini-white as a transformation marker (Figure 1A). Unlike Gateway single-fragment cloning approaches where variability is distributed between the entry clones and the destination vectors, the strategy of our MultiSite toolkit involves placing all the variability in modular entry clones. In the LR reaction, the destination vector is combined with appropriate combinations of entry clones and the distinct attL and attR sites ensure position and orientation-specific insertion of the entry clone fragments into the destination vector.

### Two-fragment Gateway MultiSite recombination cloning

A schematic diagram outlining the steps involved in two-fragment Gateway MultiSite cloning is shown in Figure 1B. As mentioned above, a major aim of this work was to generate a set of Gateway MultiSite entry clones that would be generally useful to a wide variety of researchers. For two-fragment MultiSite these entry clones include pENTR L5-GAL4-L2 and pENTR L5-QF-L2 for producing constructs with regulatory DNA upstream of GAL4 and QF, and pENTR L1-20XUAS-R5 and pENTR L1-5XQUAS-R5 for expressing genes of interest under UAS and QUAS control. The complete list of pENTR L1-R5 and pENTR L5-L2 entry clones, as well as all other entry clones described herein, is presented in Table 1 along with insert sizes, number of clones screened, and correct clones recovered for each.

**Table 1:** Entry Clones.

Entry Clones	Correct clones	Clones screened	Insert size (bp)
L1-iav-5'Reg-R5	1	1	596
L1-syjn-5'Reg-R5	5	10	3457
L1-20XUAS-R5	2	2	768
L1-5XQUAS-R5	2	2	438
L1-mCherry-T-R5	1	1	729
L1-eGFP-T-R5	1	1	738
L5-GAL4-L2	1	1	2650
L5-QF-L2	2	2	2455
L5-CHETA-YFP-L2	3	3	2289
L5-eNpHR3.0-YFP-L2	3	4	2310
L1-trpA1-5'Reg-L4	1	1	1773
L1-nompC-5'Reg-L4	1	3	4080
L1-trpL-5'Reg-L4	9	22	5968
L1-trp-5'Reg-L4	3	4	1700
L1-5XQUAS-L4	2	2	438
L1-20XUAS-L4	1	2	768
R4-GAL4-R3	1	1	2650
R4-QF-R3	2	2	2455
R4-mCherry-R3	2	2	712
R4-eGFP-R3	2	2	721
R4-n-syb-R3	3	3	547
R4-CD4-R3	3	3	1378
R4-mCherry-R3	1	1	717
R4-eGFP-T-R3	1	1	726
L3-trpA1-3'Reg-L2	1	1	3455
L3-nompC-3'Reg-L2	1	7	5143
L3-trpL-3'Reg-L2	1	1	2397
L3-trp-3'Reg-L2	1	1	2381
L3-mCherry-HA-L2	1	1	747
L3-eGFP-Myc-L2	1	1	759
L3-Rab3-L2	2	3	660
L5-mCherry-T-L4	1	1	717
L5-eGFP-T-L4	1	1	726
**Totals**	**63**	**93**	
L3-4X-mCherry-HA-L2	1	32	2910
L3-4X-eGFP-Myc-L2	3	128	2949

**Figure 2:**
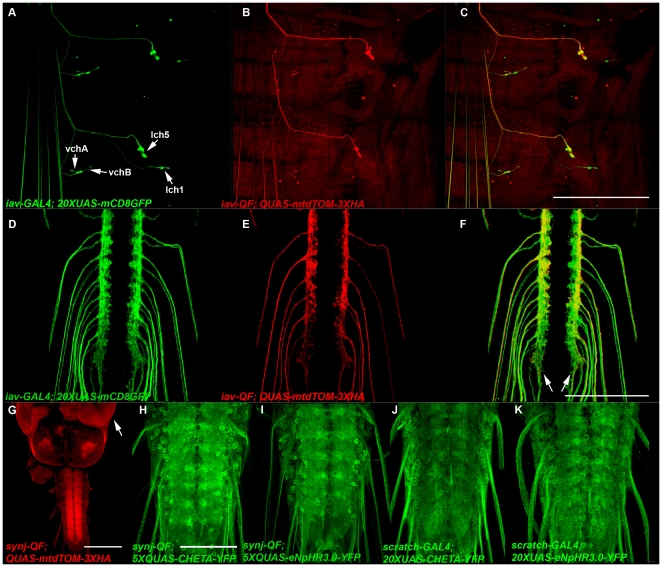
Expression of drivers and reporters generated using two-fragment Gateway MultiSite cloning. A-F) Representative confocal images of *yw*; *iav-GAL4*/*QUAS-mtdTOM-3XHA*; *iav-QF*/*20XUAS-mCD8GFP* third instar larval body wall segment (A-C) and ventral nerve cord (D-F) double-labeled with anti-GFP and anti-HA. *iav-GAL4* expresses in the chordotonal organs vchA, vchB, lch5, and lch1. *iav-QF* expresses in lch5, variably in vchA and vchB, but not in lch1. The chordotonal organ neurons of both drivers project to the lateral regions of the ventral nerve cord neuropil (arrows, F). G) Representative confocal image of *yw*; *synj-QF*/+; *QUAS-mtdTOM-3XHA*/+ third instar larva labeled with anti-HA. *synj-QF* exhibits broad expression in the nervous system, but also shows strong expression in the imaginal discs (arrow, eye-antennal disc), and moderate expression in at least the salivary gland and gut (not shown). H-K) Representative confocal images of *yw*; *synj-QF*/*5XQUAS-CHETA-YFP* (H), *yw*; *synj-QF*/*5XQUAS-eNpHR3.0-YFP* (I), *yw*; *scratch-GAL4*/*20XUAS-CHETA-YFP* (J), and *yw*; *scratch-GAL4*/*20XUAS-eNpHR3.0-YFP* (K) third instar larval ventral nerve cords labeled with anti-GFP. Each combination exhibits broad expression in the ventral nerve cord with subcellular localization observed in the cell bodies and the axo-dendritic neuropil. Scale bars: C) 500 µm, F)100 µm, G) 150 µm, H) 75 µm.

To evaluate the functionality of the pENTR L5-GAL4-L2 and pENTR L5-QF-L2 entry clones we generated pENTR L1-iav-5′Reg-R5 that contains presumptive regulatory DNA immediately upstream of the predicted translation start site of the trp ion channel family member *iav* (we use the designations 5′Reg and 3′Reg in entry clones to refer to presumptive regulatory DNA sequences upstream and downstream, respectively, of the translation start site of the gene from which they originate to distinguish them from entry clones containing protein coding sequences). pENTR L1-iav-5′Reg-R5 was combined with both pENTR L5-GAL4-L2 and pENTR L5-QF-L2 in separate LR reactions with pDESThaw to generate iav-GAL4 and iav-QF. These expression clones, as well as all other expression clones described herein, are listed in Table 2 along with total construct sizes, number of clones screened, correct clones recovered, and landing sites of the corresponding fly stocks. Confocal images of *iav-GAL4* driving the *20XUAS-CD8GFP* reporter and *iav-QF* driving the *QUAS-mtdTOM-3XHA* reporter in third instar larva are presented in Figures 2A and 2B, respectively. As shown in the figures, the *iav-GAL4* and *iav-QF* drivers exhibited a chordotonal organ-specific expression pattern consistent with the known expression of iav and it's heterodimer partner nan [6]. The patterns of expression are not identical, however, as *iav-QF* does not express in the lch1 chordotonal organ and variably expresses in the vch1 and vch2 chordotonal organs. Since the identical *iav* regulatory DNA was used for both constructs, the most likely explanation for the discrepancy is a position effect associated with the *iav-QF* landing site *attP154* that is not present with *iav-GAL4* at landing site VK00014. As shown in Figures 2D-F, the chordotonal organ neurons in which both *iav* drivers express project to the lateral regions of the ventral nerve cord (VNC) neuropil (arrows, Figure 2F).

**Table 2:** Expression Clones/Fly stocks.

Construct/stock	Correct clones	Clones screened	Construct size (kb)	Insertion site(s)
*trpA1-GAL4*	3	4	16	*VK0014*/43A
*trpA1-QF*	2	3	15.8	*attP40*/25C; *attP154*/97D
*nompC-GAL4*	1	4	19.9	*VK0014*/43A; *attP154*/97D
*nompC-QF*	2	10	19.7	*attP40*/25C; *attP154*/97D
*iav-GAL4*	2	2	11.4	*VK00014*/43A
*iav-QF*	2	3	11.2	*attP154*/97D
*trp-GAL4*	2	3	14.8	*VK00014*/43A
*trpL-QF*	6	6	18.9	*attP154*/97D
*sjn-QF*	2	2	14	*attP40*/25C
*20XUAS-CHETA-YFP*	1	3	11.2	*attP40*/25C; *attP2*/68A
*20XUAS-eNpHR3.0-YFP*	3	3	11.2	*attP40*/25C; *attP2*/68A
*QUAS-CHETA-YFP*	3	3	10.8	*attP33*/50B; *attP2*/68A
*QUAS-eNpHR3.0-YFP*	2	3	10.8	*attP33*/50B; *attP2*/68A
*QUAS-mCherry-Rab3*	1	3	9.9	*attP40*/25C; *VK00027*/89E
*QUAS-GFP-Rab3*	2	3	9.9	*attP40*/25C; *VK00027*/89E
*QUAS-N-syb-mCherry-HA*	3	3	9.8	*attP40*/25C
*QUAS-N-syb-GFP-Myc*	1	3	9.8	*attP40*/25C
*QUAS-N-syb-4XmCherry-HA*	2	4	11.9	*attP40*/25C
*QUAS-N-syb-4XGFP-Myc*	3	6	11.9	*attP40*/25C
*20XUAS-CD4-4XmCherry-HA*	3	3	12.4	*attP2*/68A
*20XUAS-CD4-4XGFP-Myc*	3	3	12.4	*attP2*/68A
**Totals**	**49**	**77**		

Since the Q system [7] was developed only recently and no QF driver exists that expresses throughout the nervous system, we attempted to develop such a driver using regulatory DNA immediately upstream of the predicted translation start site of *synaptojanin* (*synj*), a gene that functions in synaptic vesicle recycling [8]. To do this, we first generated pENTR L1-*synj*-5′Reg-R5 and subsequently used it to create *synj*-QF in an LR reaction with pENTR L5-QF-L2 and pDESThaw. *synj-QF* exhibits broad expression in the nervous system (Figure 2G), as expected, but also expresses strongly in imaginal discs (eye disc, arrow Figure 2G), and at moderate levels in at least the salivary gland and gut (not shown). Expression of *synj-QF* in non-neural tissues was not expected given neuropil-specific expression of Synj in larva has been previously reported [9]. Explanations for the discrepancy include the possibility that not all the *synj* regulatory region is included in the *synj-QF* construct or there is a position effect associated with the *attP40* insertion site.

To assess the functionality of pENTR L1-*20XUAS*-R5 and pENTR L1-*5XQUAS*-R5 we generated pENTR L5-CHETA-YFP-L2 and pENTR L5-eNpHR3.0-YFP-L2. These entry clones contain the excitatory blue light-activated sodium channel CHETA-YFP [10] and the inhibitory yellow light-activated chloride channel eNpHR3.0-YFP [11] that enable optical excitation and inhibition of neuronal activity, respectively. We subsequently used these in LR reactions with pDESThaw in all four combinations to generate *20XUAS-CHETA-YFP*, *20XUAS-eNpHR3.0-YFP*, *5XQUAS-CHETA-YFP*, and *5XQUAS-eNpHR3.0-YFP*. The expression patterns of these constructs in third instar larval ventral nerve cords using the *synj-QF* and *scratch-GAL4* drivers, which both express broadly in the larval nervous system, are shown in Figures 2H-K. All four constructs exhibit broad expression in the VNC, as expected, and both CHETA-YFP and eNpHR3.0-YFP distribute relatively evenly between the cell bodies and axo-dendritic neuropil.

### Three-fragment Gateway MultiSite recombination cloning

**Figure 3:**
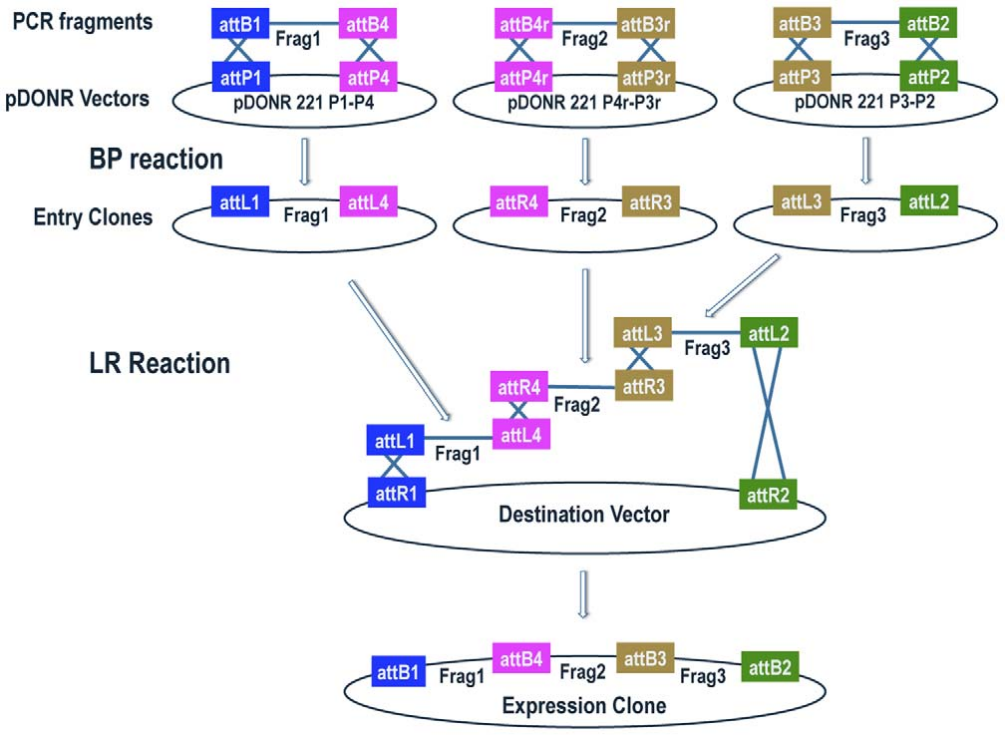
Schematic diagram of three-fragment Gateway MultiSite recombination cloning. Fragments 1, 2, and 3 are amplified by PCR using oligonucleotides that incorporate flanking attB1 and attB4 sites in fragment 1, flanking attB4r and attB3r sites in fragment 2, and flanking attB3 and attB2 sites in fragment 3. Fragment 1 is combined with pDONR 221 P1-P4, fragment 2 with pDONR 221 P4r-P3r, and fragment 3 with pDONR 221 P2-P2 in separate BP reactions. The products of the BP reactions are pENTR attL1-Frag1-attL4, pENTR attR4-Frag2-attR3, and pENTR attL3-Frag3-attL2. In the LR reaction these three entry clones are combined with a destination vector to produce an expression clone containing fragment 1, fragment 2, and fragment 3 in a position and orientation-specific manner. Note that the pENTR R4-R3 and pENTR L3-L2 entry clones are also used in four-fragment Gateway MultiSite recombination cloning. Schematic modified from the Invitrogen MultiSite Gateway Pro user manual.

A schematic diagram outlining the steps involved in three-fragment Gateway MultiSite cloning is shown in Figure 3. The entry clones generated for three-fragment MultiSite cloning expected to be of general use include pENTR R4-GAL4-R3, pENTR R4-QF-R3, pENTR L1-*20XUAS*-L4, pENTR L1-*5XQUAS*-L4, pENTR R4-mCherry-R3, pENTR R4-eGFP-R3, pENTR L3-mCherry-HA-L2, and pENTR L3-eGFP-Myc-L2.

To evaluate the functionality of pENTR R4-GAL4-R3 and pENTR R4-QF-R3 we generated entry clones containing regulatory DNA both upstream and downstream of the predicted translation start sites of several genes including *trpA1*, *nompC*, *trp*, and *trpl*. These include pENTR L1-*trpA1*-5′Reg-L4, pENTR L3-*trpA1*-3′Reg-L2, pENTR L1-*nompC*-5′Reg-L4, pENTR L3-*nompC*-3′Reg-L2, pENTR L1-*trp*-5′Reg-L4, pENTR L3-*trp*-3′Reg-L2, pENTR L1-*trpl*-5′Reg-L4, and pENTR L3-*trpl*-3′Reg-L2. These were used in the LR reactions with pDESThaw, pENTR R4-GAL4-R3 and pENTR-R4-QF-R3 to generate *trpA1-GAL4* (*trpA1*-5′Reg-GAL4-*trpA1*-3′Reg), *trpA1-QF* (*trpA1*-5′Reg-QF-*trpA1*-3′Reg), *nompC-GAL4* (*nompC*-5′Reg-GAL4-*nompC*-3′Reg), *nompC-QF* (*nompC*-5′Reg-QF-*nompC*-3′Reg), *trpl-QF* (*trpl*-5′Reg-QF-*trpl*-3′Reg), and *trp-GAL4* (*trp*-5′Reg-GAL4-*trp*-3′Reg).

**Figure 4:**
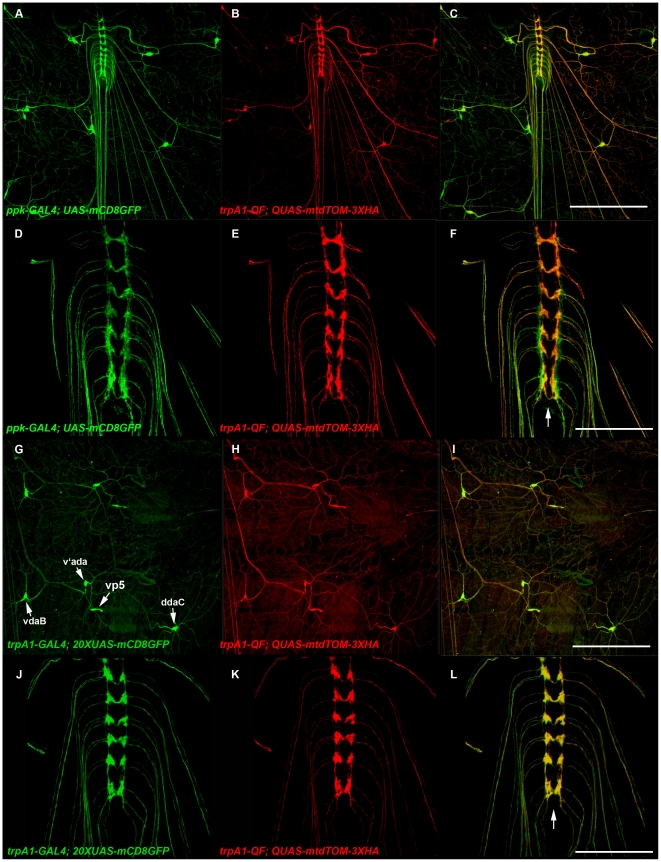
The *trpA1-GAL4* and *trpA1-QF* drivers express in type IV larval sensory neurons. A-F) Representative confocal images of *yw*; *ppk-GAL4*, *UAS-mCD8GFP*/*QUAS-mtdTOM-3XHA*; *trpA1-QF*/+ third instar larva. *trpA1-QF* exhibits overlapping expression with the class IV sensory neuron-specific driver *ppk-GAL4* (A-C). *ppk-GAL4* and *trpA1-QF* neurons project to the central region of the VNC (D-F; arrow, F). G-L) Representative confocal images of a *yw*; *trpA1-GAL4*/*QUAS-mtdTOM-3XHA*; *trpA1-QF*/*20XUAS-mCD8GFP* larva. *trpA1-GAL4* and *trpA1-QF* exhibit nearly indistinguishable patterns of expression in the three class IV neurons vdaB, v′ada, and ddaC and the external sensory organ neuron vp5 (G-I, arrow, G). *trpA1-GAL4* and *trpA1-QF* both project to the central region of the VNC (J-L, arrow, L). Both animals were double-labeled with anti-GFP and anti-HA. Scale bars: C) and I) 350 µm, F) and L) 100 µm.

The *trpA1-QF* driver exhibited expression in what appeared to be class IV larval sensory neurons based on their dendritic branching complexity [12], [13]. To determine if this was indeed the case, we coexpressed *trpA1-QF* with the class IV sensory neuron-specific driver *ppk-GAL4* and observed their expression using the *UAS-mCD8GFP* and *QUAS-mtdTOM-3XHA* reporters. As shown in Figures 4A-F, expression of *trpA1-QF* overlaps that of *ppk-GAL4*, thus confirming *trpA1-QF* expression in class IV larval sensory neurons. This expression pattern for *trpA1-QF* is consistent with expectations since *trpA1* has been previously shown to function in class IV larval sensory neurons as part of a photosensory pathway [14]. *trpA1-GAL4* expression is nearly indistinguishable from that of *trpA1-QF as* shown in Figures 4G-L. Both drivers express in the class IV larval sensory neurons vdaB, vdaa, and ddaC as well as the external sensory neuron vp5 as indicated in Figure 4G. Although the dendrites of the *trpA1* sensory neurons are distributed broadly across the larval body wall they project to a discrete region near the center of the VNC (arrow, Figure 4L). This anatomical arrangement is reminiscent of broadly distributed olfactory neurons in adult flies [15] and mice [16] that sense the same odorant and project to the same glomerulus.

**Figure 5:**
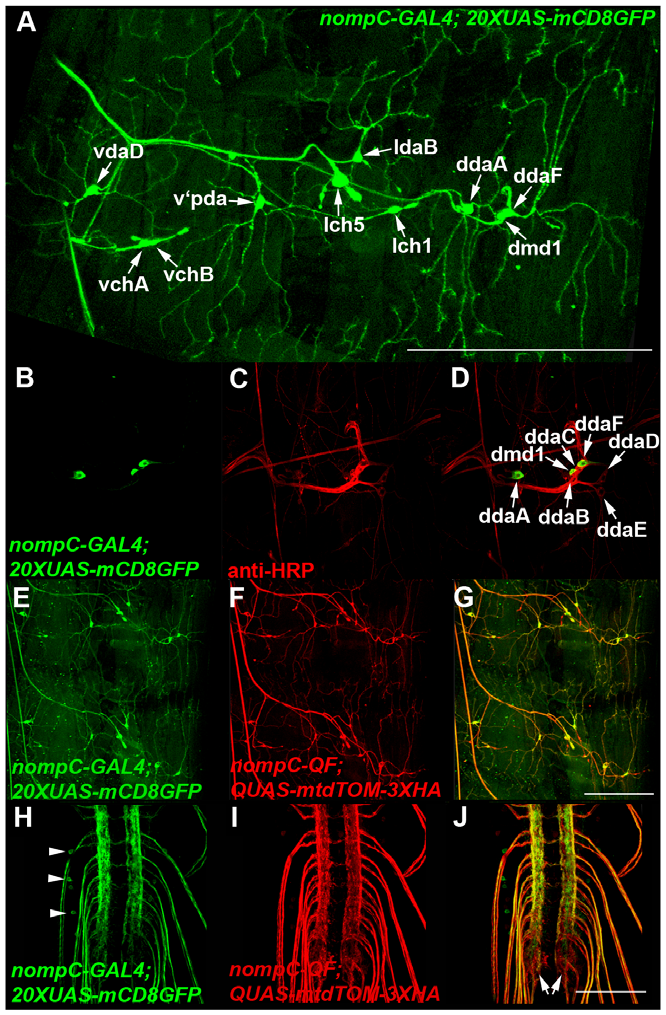
The *nompC-GAL4* and *nompC-QF* drivers express in chordotonal organs and class III larval sensory neurons. A) Representative composite confocal image of a *yw*; *nompC-GAL4*/+; *20XUAS-mCD8GFP*/+ third instar larva abdominal body wall segment labeled with anti-GFP. *nompC-GAL4* expresses in the chordotonal organs vchA, vchB, lch1 and lch5, the class III sensory neurons vdaD, v′pda, ldaB, ddaA, and ddaF, and the md neuron dmd1. B-D) Higher resolution confocal images of the dorsal cluster of sensory neurons from A) labeled with anti-GFP and anti-HRP. Expression of *nompC-GAL4* can be distinguished in ddaF, dmd1, and ddaA. E-J) Representative confocal images of a *yw*; *nompC-GAL4*/*QUAS-mtdTOM-3XHA*; *nompC-QF*/*20XUAS*-CD8GFP third instar larval body wall segment (E-G) and VNC (H-I) double labeled with anti-GFP and anti-HA. The *nompC-QF* expression pattern is very similar to *nompC-GAL4*. *nompC* sensory neurons project to the lateral regions of the VNC (arrows, J). A small number of presumptive interneurons with cell bodies in the VNC exhibit weak expression with *nompC-GAL4* (arrowheads, H). Scale bars: A) 500 µm, G) 350 µm, J) 100 µm.

The *nompC-GAL4* and *nompC-QF* drivers also express in a subset of larval sensory neurons. As shown in Figure 5A, *nompC-GAL4* expresses in the chordotonal organs vchA, vchB, lch5, and lch1, the class III sensory neurons vdaD, v′pda, ldaB, ddaA, and ddaF, and the dmd1 sensory neuron. The dorsal sensory neuron cluster from A) was double-labeled with anti-HRP and imaged at higher magnification and lower signal intensity to allow the cell body of each individual neuron to be distinguished (Figures 5B-D). As shown in Figures 5E-G, *nompC-QF* expression closely resembles that of *nompC-GAL4*. The *nompC-GAL4* and *nompC-QF* neurons both project to the lateral regions of the VNC as shown in Figures 5H-J. That these drivers express in sensory neurons is not surprising given *nompC* is a mechanoreceptor known to function in sensory perception [17], [18]. Not unlike the class IV neurons, the class III and chordotonal organ neurons are broadly distributed across the larval body wall, but project to discrete regions of the VNC (different from class IV neurons). These observations suggest a consolidation of sensory information type in second-order neurons, again, similar to previous observations in the olfactory neurons of the adult fly and mice.

**Figure 6:**
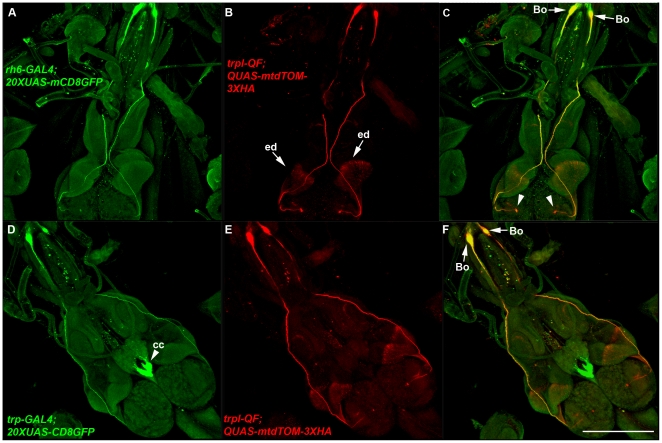
The *trpl-QF* and *trp-GAL4* drivers express in Bolwig's organ larval photoreceptors. A-C) Representative confocal images of a *yw*; *trpl-QF*/*20XUAS-mCD8GFP*; *Rh6-GAL4*/ *QUAS-mtdTOM-3XHA* third instar larva. *trpl-QF* exhibits overlapping expression with *Rh6-GAL4* in Bolwig's organ. Bolwig's organ photoreceptor cell bodies are indicated (arrows, C). These photoreceptor neurons project to the central region of the larval brain hemispheres (arrowheads, C). In addition, *trpl-QF* expresses in the eye imaginal disc (arrows, B). D-F) Representative confocal images of a *yw*; *trp-GAL4*/*QUAS-mtdTOM-3XHA*; *trpl-QF*/*20XUAS-mCD8GFP* third instar larva. *trp-GAL4* exhibits overlapping expression with *trpl-QF* in Bolwig's organ photoreceptors (arrows, F). *trp-GAL4* also exhibits strong expression in the corpus cardiacum region of the ring gland (arrowhead, D). Both images were double-labeled with anti-GFP and anti-HA. Bo-Bolwig's organ; cc-corpus cardiacum; ed-eye imaginal disc. Scale bar: 350 µm.

The *trpl-QF* driver exhibits expression in neurons at the anterior end of the larva whose cell bodies reside adjacent to the larval mouth hooks. These neurons seemed most likely to be the photosensitive Bolwig's organ neurons both because of their anatomical location and also because it would not be surprising if *trpl* functioned in larval phototransduction since its function in adult phototransduction is well established [19]. To determine if the neurons in which *trpl-QF* expresses are Bolwig's organ photoreceptors, it was co-expressed with the Bolwig's organ driver *Rh6-GAL4* and both were visualized using the *20XUAS-mCD8GFP* and *QUAS-mtdTOM-3XHA* reporters. As shown in Figures 6A-C, *trpl-QF* expression overlaps with *Rh6-GAL4* expression in Bolwig's organ neurons (arrows, Figure 6C) whose projections terminate near the center of the larval brain hemispheres (arrowheads, Figure6C). The *trpl-QF* driver also exhibits expression in the eye imaginal disc (arrows, Figure 6B).

The *trp-GAL4* driver also expresses in Bolwig's organ neurons as determined by its overlapping expression with *trpl-QF* (Figures 6D-F) when the two were coexpressed and visualized using the reporters *20XUAS-mCD8GFP* and *QUAS-mtdTOM-3XHA*. Like *trpl*, this is not surprising since the role of *trp* in adult phototransduction is also well established [19]. Unlike *trpl-QF*, expression of *trp-GAL4* was not observed in the eye imaginal disc. Surprisingly, *trp-GAL4* exhibited its strongest expression in the corpus cardiacum (arrowhead, Figure 6D), a component of the larval ring gland that is believed to mediate neuroendocrine functions. Since the corpus cardiacum is known to function in sensing and regulating glucose levels in the larva [20], *trp* expression in this region of the ring gland raises the intriguing possibility that *trp* has been co-opted by the corpus cardiacum to transduce signals associated with glucose sensing in larva, not unlike its established role of tranducing signals in adult flies associated with photoreception (or vice versa, depending on which role came first evolutionarily).

To evaluate the functionality of pENTR L1-*5XQUAS*-L4, R4-mCherry-R3, R4-GFP-R3, L3-mCherry-HA-L2, and L3-GFP-Myc-L2, we decided to attempt to generate red and green fluorescent synaptic vesicle (SV) markers for the Q system since no SV markers for it currently exist. The approach we took was to fuse the fluorescent proteins to the synaptic vesicle membrane-associated proteins Rab3 and neuronal-synaptobrevin (*n-syb*) and place them under QUAS control. To do this we generated the entry clones pENTR L3-Rab3-L2 and pENTR R4-n-syb-R3 which contain the protein coding sequences of *Drosophila* Rab3 and n-syb [21]. We combined these entry clones in the LR reaction with pDESThaw in various combinations to generate 5XQUAS-eGFP-Rab3, *5XQUAS-mCherry-Rab3*, *5XQUAS-n-syb-eGFP-Myc*, and *5XQUAS-n-syb-mCherry-HA*.

**Figure 7:**
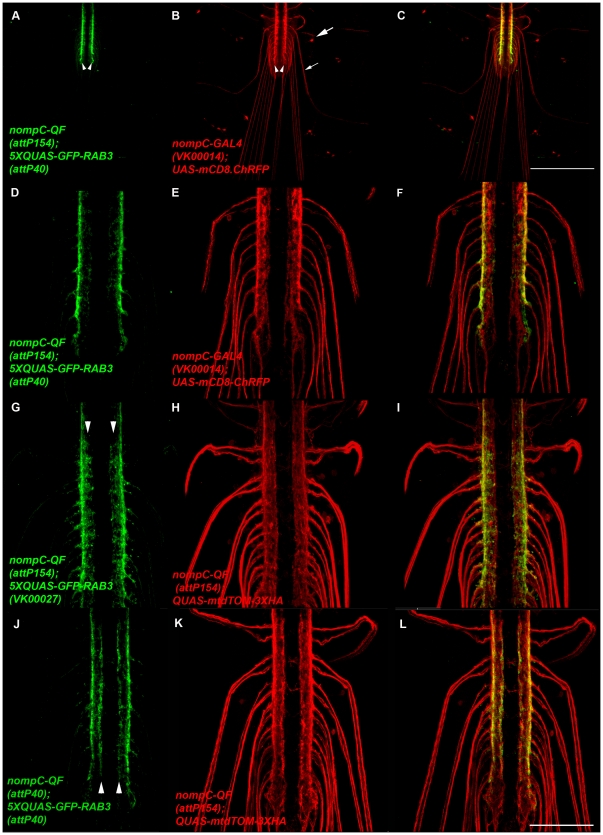
*5XQUAS-GFP-Rab3* is a green fluorescent reporter of synaptic vesicles for the Q system. A-F) Representative confocal images of a *yw*; *nompC-GAL4*/*5XQUAS-GFP-Rab3* (*attP40*); *nompC-QF* (*attP154*)/*UAS-mCD8.ChRFP* third instar larva double-labeled with anti-GFP and anti-CD8. mCD8.ChRFP localizes to the cell body (large arrow, B), axon (small arrow, B), and lateral neuropil (arrowheads, B) regions of *nompC* neurons. GFP-Rab3 localization is only observed in the lateral neuropil region of the ventral nerve cord (arrowheads, A) where the presynaptic terminals of *nompC* neurons are located. The image in A) was collected at higher than usual signal intensity to detect possible weak signals in the cell body or axonal regions but none were observed. D-F) Higher magnification images of the ventral nerve cord. G-I) Representative confocal images of a *yw*; *QUAS-mtdTOM-3XHA*/+; *nompC-QF* (*attP154*)/*5XQUAS*-GFP-Rab3 (VK00027) third instar larva ventral nerve cord. The VK00027 insertion site of *5XQUAS*-GFP-Rab3 exhibits increased expression levels relative to *attP40* in the more medial portion of the *nompC* neuron projection region (arrowheads, G). J-L) Representative confocal images of a *yw*; *nompC-QF* (*attP40*)/*5XQUAS*-*GFP-Rab3* (*attP40*); *QUAS-mtdTOM-3XHA*/+ third instar ventral nerve cord. The *attP40* insertion site of *nompC-QF* exhibits increased expression levels relative to *attP154* in the more medial portion of the *nompC* neuron projection region (arrowheads, J). The images in G-L were double-labeled with anti-GFP and anti-HA. Scale bar: 100 µm.

To assess expression and localization of *5XQUAS-GFP-Rab3* it was co-expressed with the *UAS-mCD8.ChRFP* reporter using the *nompC-QF* and *nompC-GAL4* drivers described above. As shown in Figure 7A, GFP-Rab3 localization was only detectable in the region of the VNC where the *nompC* neuron presynaptic terminals are located (arrowheads). In contrast, as shown in Figure 7B, mCD8.ChRFP distribution was observed throughout the *nompC* neurons including the cell bodies (large arrow), axons (small arrow), and presynaptic terminals (arrowheads). Higher resolution views of the VNC region are shown in Figures 7D-F. A similar experiment was performed using *5XQUAS-mCherry-Rab3* and *20XUAS-mCD8GFP* except observation was via direct fluorescence and similar results were obtained (data not shown). Restricted localization of both GFP-Rab3 and mCherry-Rab3 to the presynaptic terminal regions of *nompC* neurons indicates both *5XQUAS-eGFP-Rab3* and *5XQUAS-mCherry-Rab3* are reliable SV reporters for the Q system. Similar constructs were made with fusions of eGFP and mCherry to the carboxy-terminus of Rab3, but these were weakly expressed and did not localize to presynaptic terminals (data not shown). This result indicates Rab3 localization to SVs will not tolerate carboxy-terminal fusions of these fluorescent proteins.

**Figure 8:**
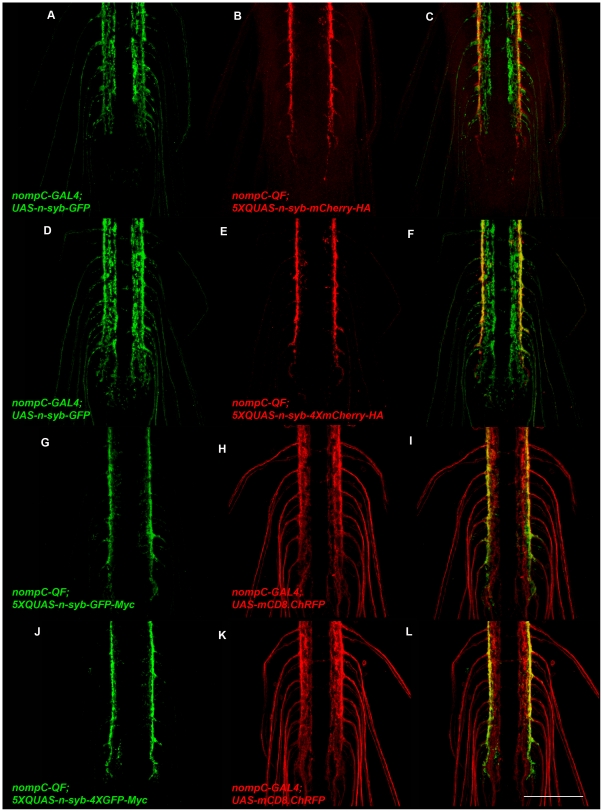
Fusion variants of neuronal-synaptobrevin function as red and green fluorescent synaptic vesicle reporters for the Q system. A-F) Representative confocal images of *yw*; *nompC-GAL4*/*5XQUAS*-n-syb-mCherry-HA; *nompC-QF* (*attP154*)/UAS-n-syb-GFP (A-C) and *yw*; *nompC-GAL4*/*5XQUAS-n-syb-4XmCherry-HA*; *nompC-QF* (*attP154*)/*UAS-n-syb-GFP* (D-F) third instar larval ventral nerve cords double-labeled with anti-GFP and anti-HA. Expression of n-syb-mCherry-HA and n-syb-4XmCherry-HA is restricted to the lateral regions of the neuropil where *nompC* neuron presynaptic terminals are located. G-L) Representative confocal images of *yw*; *nompC-GAL4*/*5XQUAS-n-syb-GFP-Myc* (G-I) and *yw*; *nompC-QF* (*attP154*)/*UAS-mCD8.ChRFP* third instar larval ventral nerve cords double-labeled with anti-GFP and anti-CD8. Expression of n-syb-GFP-Myc and n-syb-4XGFP-Myc is restricted to the lateral regions of the neuropil where *nompC* neuron presynaptic terminals are located. Scale bar: 100 µm.

The expression and localization of *5XQUAS-n-syb-mCherry-HA* and *5XQUAS-n-syb-GFP-Myc* was assessed by co-expression with *UAS-n-syb-GFP* and *UAS-mCD8.ChRFP*, respectively, also using the *nompC-QF* and *nompC-GAL4* drivers described above. Similar to GFP and mCherry-tagged Rab3, n-syb-mCherry-HA and n-syb-GFP-Myc were not detectable an*yw*here in the *nompC* neurons except the presynaptic terminal regions as shown in Figures 8A-C, G-I. These observations indicate both *5XQUAS-n-syb-mCherry-HA* and *5XQUAS-n-syb-GFP-Myc* are also reliable SV reporters for the Q system. Although n-syb-mCherry-HA was detected with the anti-HA antibody 3F10 (Roche), we were unable to detect n-syb-eGFP-Myc with any of three anti-Myc antibodies (9E10-Developmental Studies Hybridoma Bank; 4A6-Millipore; 289-19510-Invitrogen/Molecular Probes) even though all three successfully labeled a different positive control myc-tagged protein in larval immunohistochemistry (data not shown). This suggests the myc-tag epitopes recognized by these antibodies are not accessible, but it does not preclude the possibility that this will be the case for all anti-myc antibodies.

Although no expression of any of the SV markers was observed outside the presynaptic terminal regions of *nompC* neurons, their expression did not extend as far medially as the other reporters (Figures 7C,F and 8C,I). One potential reason for this is a position effect of the *nompC-QF* driver at landing site *attP154*. To test this possibility, the same *nompC-QF* construct was inserted at landing site *attP40* and used to express the same *5XQUAS-GFP-Rab3* as before inserted at the *attP40* landing site. As shown in Figure 7G, expression is observed in the more medially projecting *nompC* neurons (arrowheads) in this combination, suggesting the lack of expression of the same reporter in these neurons with *nompC-QF* at *attP154* is due to a position effect.

**Figure 9:**
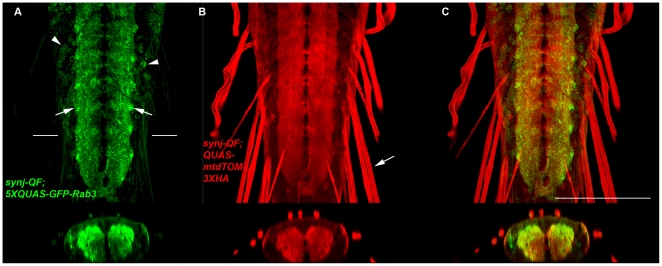
GFP-Rab3 distributes broadly within the larval neuropil when driven by *synj-QF*. A-C) Representative confocal images of *synj-QF*/*5XQUAS-GFP-Rab3* (*attP40*); *QUAS-mtdTOM-3XHA*/+ third instar larval ventral nerve cords double-labeled with anti-GFP and anti-HA. GFP-Rab3 localization is highly preferential to the neuropil region of *synj*-QF neurons (arrows, A) and is broadly distributed within the neuropil similar to mtdTOM-3XHA. However, a small amount of GFP-Rab3 is detected in the cell bodies (arrowheads, A) that that is probably a result of the high level of expression of syjn-QF. In contrast to mtdTOM-3XHA that distributes robustly to axons and dendrites within larval nerves (arrow, B), GFP-Rab3 is nearly undetectable in these regions of larval sensory and motor neurons. Bottom panels are orthogonal views of the images above. White bars in A) indicate the location from which the orthogonal views were taken. Scale bar: 100 µm.

Expression of *5XQUAS-GFP-Rab3* was also examined using the *synj-QF* driver described above. The results of co-expression with *QUAS-mtdTOM-3XHA* are shown in Figures 9A-C. The majority of GFP-Rab3 localizes to the neuropil region (arrows, A) where synaptic vesicles are located. In contrast, expression of mtdTOM-3XHA is observed not just in the neuropil, but also in the nerves containing axons and dendrites (arrow, B). In these larvae, low levels of GFP-Rab3 were detected in the cell bodies of VNC neurons (arrowheads, A). This is probably due to higher levels of expression of GFP-Rab3 per neuron using *synj-QF* as compared to *nompC-QF* such that the higher levels of expression with *synj-QF* has slightly exceeded the capacity of the cellular machinery to localize GFP-Rab3 to synaptic vesicles. Thus, expressing these SV reporters at high levels may reduce their reliability as SV reporters.

### Four-fragment Gateway MultiSite recombination cloning

**Figure 10:**
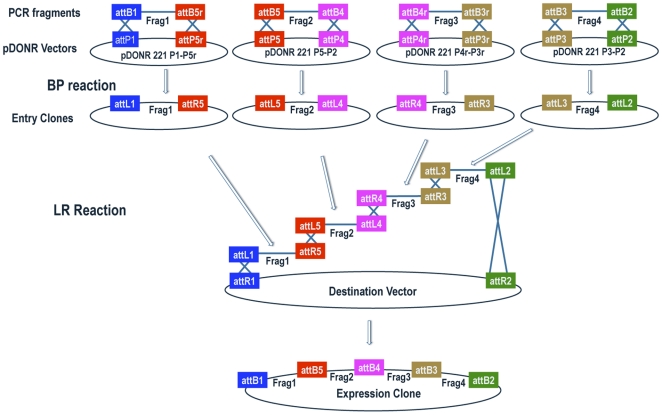
Schematic diagram of four-fragment Gateway MultiSite recombination cloning. Fragment 1, fragment 2, fragment 3, and fragment 4 are amplified by PCR using oligonucleotides that incorporate flanking attB1 and attB5r sites in fragment 1, flanking attB5 and attB4 sites in fragment 2, flanking attB4r and attB3r sites in fragment 3, and flanking atB3 and attB2 sites in fragment 4. Fragment 1 is combined with pDONR 221 P1-P5r, fragment 2 with pDONR 221 P5-P4, fragment 3 with pDONR 221 P4r-P3r, and fragment 4 with pDONR 221 P3-P2 in separate BP reactions. The products of the BP reactions are pENTR attL1-Frag1-attR5, pENTR attL5-Frag2-attL4, pENTR attR4-Frag3-attR3, and pENTR attL3-Frag4-attL2. In the LR reaction these four entry clones are combined with a destination vector to produce an expression clone containing fragment 1, fragment 2, fragment 3, fragment 4 in a position and orientation-specific manner. Note that pENTR L1-R5 entry clones are used in two-fragment and pENTR R4-R3 and pENTR L3-L2 entry clones in three-fragment Gateway MultiSite recombination cloning. Schematic modified from the Invitrogen MultiSite Gateway Pro user manual.

A schematic diagram of four-fragment Gateway MultiSite recombination cloning is shown in Figure 10. While there is no overlap in the usage of entry clones between two and three-fragment Gateway MultiSite cloning, the pENTR L1-R5 (first position, two-fragment cloning), pENTR R4-R3 (second position, three-fragment cloning), and pENTR L3-L2 (third position, three-fragment cloning) entry clones listed in Table 1 can also be used in four-fragment cloning.

To test the functionality of four-fragment Gateway MultiSite recombination cloning we made constructs containing four tandem copies of either eGFP or mCherry. This required the generation of the entry clones pENTR L1-eGFP-T-R5, pENTR L5-eGFP-T-L4, and pENTR R4-eGFP-T-R3 and the analogous entry clones for mCherry (T is for tandem to distinguish their intended purpose). These entry clones contain neither start nor stop codons and were used in appropriate combinations with pENTR L3-eGFP-Myc-L2 and pENTR L3-mCherry-HA-L2 (described above) and pDESThaw to generate 4X-GFP-Myc and 4X-mCherry-HA. These constructs are not functional in isolation, since, in addition to the absence of start codons, they have no upstream UAS or QUAS regulatory regions. Our intention with them was to use them as PCR templates and incorporate them into pENTR L3-L2 entry clones containing four tandem repeats of either eGFP or mCherry that could then be used in subsequent LR reactions. The difficulty in generating these entry clones was that in the second round BP reactions there were two competing attB3 sites: an internal attB3 site (a product of the first round LR reaction) and the flanking attB3 site. We recovered colonies utilizing the desired flanking attB3 site (and thus containing all four tandem repeats of GFP or mCherry) at a frequency of approximately one in 40 relative to utilization of the internal attB3 site as indicated at the bottom of Table 1.

The L3-4XmCherry-HA-L2 and L3-4XeGFP-Myc-L2 entry clones were then used in combination with pENTR L1-*5XQUAS*-L4 and pENTR R4-n-syb-R3 to generate *5XQUAS-n-syb-4XGFP-Myc* and *5XQUAS-n-syb-4XmCherry-HA*. Expression of *5XQUAS-n-syb-4XmCherry-HA* and *5XQUAS-n-syb-4XGFP-Myc* were compared to *UAS-n-syb-GFP* and *UAS-mCD8.ChRFP*, respectively, using *nompC-QF* and *nompC-GAL4*. As shown in Figures 8D-F, J-L, n-syb-4XmCherry-HA (Figure 8E) shows a nearly indistinguishable distribution pattern from n-syb-mCherry-HA (Figure 8B). Similarly, n-syb-4XeGFP-Myc (Figure 8J) exhibits a nearly indistinguishable distribution pattern from n-syb-eGFP-Myc (Figure 8G). These results indicate that having four tandem copies of either eGFP or mCherry fused to the carboxy-terminus of n-syb does not alter its SV localization as compared to fusion of a single copy of eGFP or mCherry. However, whether this will be the case for other proteins can only be determined empirically.

To determine if there were differences in fluorescence intensity between n-syb fused to one vs four copies of mCherry or eGFP, direct fluorescence images of age-matched third instar larva containing n-syb fused to one or four copies of mCherry or eGFP under QUAS control and expressed using the same *nompC-QF* driver were acquired using identical microscope settings between comparison genotypes. Representative, unmodified confocal images of third instar larval VNCs are shown in Figures S2A-D. Quantification revealed 3.5X +/- 0.73 (std dev; n = 3) higher fluorescence intensity for n-syb fused to four copies of mCherry as compared to one, and 4.4X +/- 1.9 (std dev; n = 3) higher fluorescence intensity for n-syb fused to four copies of eGFP as compared to one. Both of these values are consistent with the 4X expectation. That all four copies of mCherry are translated in n-syb-4XmCherry-HA was demonstrated by its detection of the HA epitope tag at its carboxy-terminus with an anti-HA antibody and an Alexa488 coupled secondary antibody whose green fluorescent signal could not be mistaken for direct mCherry fluorescence (data not shown). Similar to n-syb-eGFP-Myc, n-syb-4XeGFP-Myc was not detected with any of the three anti-Myc antibodies mentioned above, so it was not possible to confirm all four copies of eGFP are translated using this approach, but the 4.4X higher fluorescence intensity supports this conclusion.

Given our success in fusing four tandem repeats of the mCherry and eGFP fluorescent proteins to the synaptic vesicle-specific protein n-syb and getting the expected ∼4X increase in fluorescence intensity without altering its synaptic vesicle localization suggests this might be a general strategy for increasing the fluorescence signal of other subcellular markers. This could be particularly useful in cases where it is not feasible to boost the fluorescence signal of a fluorescence-tagged subcellular marker by further increasing the expression level of the protein because the capacity of the cell to properly localize the protein to its subcellular compartment has already reached saturation and further increases in expression would compromise the reliability of the marker. By fusing tandem repeats of fluorescent proteins to subcellular marker proteins, the quantity of fluorescence per protein molecule can be increased (apparently linearly in our example), and thus the reliable range of subcellular marker fluorescence signal can be increased beyond what would be possible with fusion of a single fluorescent protein to a given subcellular marker.

**Figure 11:**
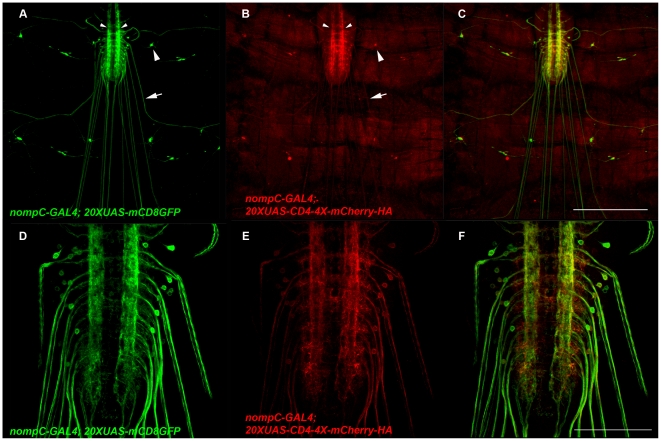
The CD4-4XmCherry-HA reporter preferentially localizes to presynaptic terminals. A-F) Representative confocal images of *yw*; *nompC-GAL4*/*20XUAS-hCD4-4XmCherry-HA*; *20XUAS-mCD8GFP*/+ third instar larva double-labeled with anti-GFP and anti-HA. A-C) mCD8-GFP is easily visualized in cell bodies (larger arrowhead, A), axons (arrow, A) and presynaptic terminals (smaller arrowheads, A). In contrast, CD4-4X-mCherry-HA (B) is barely visible in the cell bodies and axons, but exhibits preferential localization to presynaptic terminals (labeling same as in A). D-F) Higher magnification images of the VNC. Scale bars: C) 350 µm, F) 100 µm.

To test the functionality of pENTR L1-*20XUAS*-L4, the entry clone pENTR R4-hCD4-R3 (containing full-length human CD4) was generated and used with pENTR L3-4XmCherry-HA-L2 and pDESThaw in an LR reaction to produce the expression clone *20XUAS*-hCD4-4XmCherry-HA. Since CD4, like CD8, is a transmembrane protein, it was hoped that this construct would distribute throughout the plasma membrane of any cell expressing it and thus provide an alternative reporter of cell morphology of similar utility as the widely used mCD8GFP. *nompC-GAL4* was used to co-express *20XUAS-hCD4-4XmCherry-HA* and *20XUAS-mCD8GFP* in third instar larva and the results are shown in Figures 11A-F. In Figure 11A, mCD8GFP localizes to cell bodies (large arrowhead), axons (arrow), and the lateral neuropil (small arrowheads) as previously observed. In contrast, hCD4-4XmCherry-HA is barely visible in cell bodies or axons, but exhibits a marked preference for presynaptic terminals. Higher magnification images of the VNC are shown in Figures 11D-F. Similar results were obtained with hCD4-mCherry-HA containing only a single copy of mCherry (data not shown). Since hCD4-4XmCherry-HA does not distribute to the plasma membrane with the same uniformity as mCD8GFP, it may therefore be of limited use, but the functionality of pENTR L1-*20XUAS*-L4 is nevertheless demonstrated.

## Discussion

In this report we presented and verified the functionality of a collection of entry clones and a *Drosophila* destination vector for Gateway MultiSite recombination cloning. This modular toolkit enhances the efficiency and flexibility with which DNA constructs can be generated and it therefore represents a significant advance over Gateway single-fragment cloning, and an even greater advance over traditional restriction enzyme-based cloning. The series of entry clones we developed can be used in two, three, and four fragment MultiSite cloning to produce a wide variety of expression constructs as we have demonstrated in *Drosophila*. They can, however, potentially be utilized in other model systems pending only the development of suitable destination vectors.

### Advantages of Gateway MultiSite cloning

Single-fragment Gateway cloning is advantageous over traditional restriction enzyme-based cloning because the frequency of background colonies containing incorrect clones is sharply lower, the recombination reactions are more efficient and reliable than ligations, and internal recombinase recognition sequences are essentially nonexistent (and therefore don't have to be considered on a case-by-case basis in cloning strategies). Thus, single-fragment Gateway cloning results in an increase in cloning efficiency in certain instances and is especially suitable for high-throughput, repetitive cloning applications.

However, single-fragment Gateway cloning has the disadvantage that destination vectors are difficult to construct. They are typically generated using traditional restriction enzyme cloning, entail multiple cloning steps, and require a special bacterial strain that suppresses the lethality of the ccdB gene in the Gateway cassette. Thus, the decision to use traditional restriction enzyme cloning or Gateway cloning usually hinges on the expected frequency of use of the destination vector. For many individual labs not engaged in highly repetitive genome scale projects, it is thus often not worth the up-front time investment to generate destination vectors if they will only be used once or infrequently. Hence, the majority of cloning is still restriction enzyme-based.

The Gateway MultiSite cloning toolkit presented here has the same advantages mentioned above over restriction enzyme-based cloning as single-fragment Gateway cloning, but it does not have the disadvantage that it requires the construction of large numbers of destination vectors. In Gateway single-fragment cloning, most variations, such as fluorescent or epitope tags, etc., are incorporated in destination vectors. Hence, single-fragment Gateway cloning systems necessitate the construction of large numbers of destination vectors incorporating each novel variation. As examples, 66 destination vectors were generated in one such *Drosophila* system [22] and 288 destination vectors in a yeast system [23]. In contrast, introducing a new variant into the Gateway MultiSite toolkit described here only requires the construction of a new entry clone. As already mentioned above, destination vectors are typically generated through a time-consuming, multi-step restriction enzyme-based cloning process. Since up to four DNA fragments can be simultaneously cloned into a destination vector using Gateway MultiSite recombination cloning, this allows variations to reside in entry clones that are easily generated in an efficient, single step process. This is a major advantage over single-fragment Gateway cloning approaches because it obviates the need to generate large numbers of destination vectors. Since the up-front investment for using Gateway MultiSite cloning is thus largely reduced from generating destination vectors to generating entry clones, this should make it an appealing alternative to restriction enzyme cloning even for the routine, non-repetitive day-to-day cloning performed by the vast majority of research labs. The strategic, modular design of this Gateway MultiSite toolkit allowed the generation by two, three, and four-fragment Gateway MultiSite cloning of all of the wide variety of expression constructs presented using a single destination vector.

Furthermore, since entry clones have the versatility to be used with any compatible destination vector they eliminate the need to generate destination vectors for all model systems with a compatible destination vector. For example, if a brighter, more photostable version of GFP were developed, to incorporate the new version using single-fragment Gateway based systems would require the construction of new destination vectors for each model system. In contrast, the generation of a single entry clone containing the new GFP variant would be sufficient to allow any model system with a compatible destination vector to utilize it.

### Limitations of Gateway MultiSite cloning

Gateway MultiSite recombination cloning allows the insertion of two, three, or four fragments into a destination vector to generate an entry clone. This flexible capacity will be sufficient for a broad range of cloning applications, especially if common elements are strategically integrated into the destination vectors as was done with the *Drosophila* destination vector pDESThaw. However, for some applications insertion of four fragments will not be sufficient. In these cases, there are several options to overcome the four-fragment limitation. One option is to fuse fragments together using hybrid PCR, or other method of fusion such as Invitrogen's GENEART or Clonetech's In-Fusion systems, before incorporation into entry clones. This option may also be useful if the linker region between two fragments in the expression clone cannot be tolerated. If the linker region is not translated this is less likely to be a problem, but if it is translated as part of a fusion protein then the potential for intolerance is increased. A second option is to do sequential Gateway MultiSite cloning such that products of a first round LR reaction containing multiple elements are cloned into an entry clone that can then be used in a second round LR reaction. Although difficult due to the presence of internal attB sites in the second round BP reaction, this approach is possible as we demonstrated with the generation of the pENTR L3-4XeGFP-Myc-L2 and pENTR L3-4XmCherry-HA-L2 entry clones that contain four tandem copies of GFP or mCherry, respectively. A third option is to incorporate additional elements into destination vectors.

What are the size limitations for entry and expression clones using MultiSite Gateway cloning? The largest fragment we successfully inserted into an entry clone was 5968bp to create pENTR L1-*trpl*-5′Reg-L4. We made several attempts to clone PCR fragments ranging in size from 7–11kb into entry clones but were not successful even when the BP reactions were allowed to proceed overnight. These results demonstrate it is possible to produce entry clones with inserts ∼6 kb in size, but suggests generating entry clones with larger insert sizes will not be routine.

Five expression clones were generated by the LR reaction that were over 15 kb in total size with the largest of these being nompC-GAL4 at 19.9 kb. As noted in the Materials and Methods, the combined success frequency (percentage of colonies containing the correct clone) was 51.8% for these five constructs. This relatively high frequency of recovering correct expression clones in the 15–20 kb size range suggests the expression clone size limit using Gateway MultiSite cloning has not been reached. In instances where the LR reactions failed to produce the correct expression clone with high frequency, it was typically due to degraded or poorly quantitated input DNAs and repeats of the LR reactions using fresh DNA preps were usually successful.

While Gateway MultiSite cloning has several advantages over restriction enzyme cloning as discussed above, cost must also be a consideration. Unfortunately, the initial cost of the Gateway MultiSite kit containing the six essential pDONR vectors is substantial and will be a factor limiting the adoption of Gateway MultiSite cloning. However, once the one-time cost of the six pDONR vectors has been borne the ongoing cost of the BP and LR enzyme mixes is not that out of line with the cost of restriction enzymes and ligase, especially if the BP and LR reactions are carried out at half the volumes of that recommended by the manufacturer as we did. Also, because of the high efficiency of the BP and LR reactions there is likely to be some cost savings over restriction enzyme cloning in reagents used for preparation of miniprep DNA since correct clones are recovered at high frequency.

### Concluding remarks

This Gateway MultiSite recombination cloning toolkit provides a basic set of entry clones for potential use in any model system and a compatible destination vector for *Drosophila* transgenesis. The utility of the toolkit will only expand as additional entry clones and destination vectors become available. To facilitate use of the toolkit, we have established a website database to inventory entry clones and destination vectors compatible with Gateway MultiSite recombination cloning (www.gatewaymultisite.org).

## Materials and Methods

### Molecular biology

PCR was performed using AccuPrime *Pfx* SuperMix (Invitrogen-Cat. # 12344-040). The template for all genomic PCR reactions was genomic DNA isolated from the strain *y; cn bw sp* [24]. The templates for protein-coding entry clones were as follows: L5-CHETA-EYFP-L2⇒pLenti-CaMKIIa-hChR2(E123T-H134R)-EYFP [10], L5-eNpHR3.0-EYFP-L2⇒pLenti-CaMKIIa-eNpHR 3.0-EYFP [11], L5-QF-L2 and R4-QF-R3⇒pattB-QF-SV40 [7], L1-*5XQUAS*-R5 and L1-*5XQUAS*-L4⇒pQUAST [7], L1-*20XUAS*-R5 and R4-*20XUAS*-R3⇒pJFRC7 [25], L5-GAL4-L2 and R4-GAL4-R3⇒CaSpeR-AUG-GAL4 [15], L3-Rab3-L2 ⇒Rab3 cDNA (BDGP LP05860), R4-n-syb-R3⇒ *n-syb* cDNA [21], R4-CD4-R3⇒pMX hCD4 [26], R4-eGFP-R3 L3-eGFP-Myc-L2, L1-eGFP-T-R5, L5-eGFP-T-L4, and R4-eGFP-T-R3⇒pEGFP (Clontech Cat# 6077-1), R4-mCherry-R3, L3-mCherry-HA-L2, L1-mCherry-T-R5, L5-mCherry-T-L4, and R4-mCherry-T-R3⇒ pmCherry [27] (Clontech Cat. #-632522). Primer sequences are available upon request. All entry clones containing protein-coding genes were sequenced to ensure errors were not introduced by PCR. All entry clones containing genomic DNA were sequenced on both ends and exhibited the predicted restriction patterns.

The pDESThaw vector was constructed in four steps. First, a 0.4 kb *Nhe I*/*Asc I* PCR fragment containing the *PhiC31* attB site from pBPGUw [4] was cloned into the *Nhe I*/*Asc I* sites of pCasPeR5 [28]. Second, a 0.25 kb *Xba I*/*Bam HI* PCR fragment containing the hsp70 polyadenylation sequence from pBPGUw was cloned into the *Xba I*/*Bam HI* sites of the resulting vector from step 1. Third, the 1.7 kb Gateway cassette RF C.1 (Invitrogen Cat # 11828-029) was cloned into the *Hpa I* site of the resulting vector from step two. The resulting vector from step three we designate pDESThawP. This vector is a functional destination vector that contains both P-element ends for P-element-mediated transformation as well as the *PhiC31* attB site for *PhiC31* integrase-mediated transformation but it was not used in this study. Fourth, to eliminate the P-element ends, the 6.7 kb *Nsi I*/*Asc I* fragment from pDESThawP was cloned into the *Pst I*/*Asc I* sites of pBS-C5 [29] to generate pDESThaw which was used exclusively in this study.

The six pDONR vectors were obtained as part of the MultiSite Gateway Pro Plus kit (Invitrogen-Cat # 12537-100). All BP reactions were performed using the BP Clonase II enzyme mix (Invitrogen-Cat # 11789-020) in 5 µl volumes (half the manufacturer's recommendation) for 1 hr to overnight at room temperature (Proteinase K digestion omitted), run through a spin column (Zymo Research Cat # D4104), eluted in 6 µl of water, electroporated (2 µl) into 50 µl *DH10B* electrocompetent cells prepared in our laboratory, and plated on LB kanamycin plates (50 µg/ml).

The LR reactions were performed using the LR Clonase II Plus enzyme mix (Invitrogen-Cat # 12538-120) in 5 µl volumes (half the manufacturer's recommendation) overnight at room temperature (Proteinase K digestion omitted), run through a spin column (Zymo Research Cat # D4104), eluted in 6 µl of water, electroporated (2 µl) into 5 µl ElectroMax *DH10B* electrocompetent cells (one-fourth the manufacturer's recommendation; Invitrogen-Cat # 18290-015) +45 µl ice-cold sterile water, and plated on LB carbenicillin plates (50 µg/ml).

Quantitative data on the efficiencies of the BP and LR reactions is shown in Tables 1 and 2, respectively. Overall efficiency (percent of colonies containing correct clones) for the BP reactions used to generate 33 of the 35 entry clones described herein was determined to be 67.7% (63/93) (L3-4X-mCherry-HA-L2 and L3-4X-eGFP-Myc-L2 were excluded from this calculation because they were generated in sequential BP reactions that had competing attB3 sites as described in Results). We also note a size dependency to the success rate of the BP reactions. For the 28 BP reactions where the insert size was <3 kb the success rate was 92% (46/50), while for the five BP reactions where the insert size was >3 kb the success rate was 39.5% (17/43). The overall success rate for the 21 LR reactions was determined to be 63.6% (49/77). The success rate of the LR reactions also decreases with size although it is less pronounced than for the BP reactions. For the 16 LR reactions where the total size of the final construct was <15 kb the success rate was 70% (35/50), while for the 5 LR reactions where the total size of the final construct was >15kb it was 51.8% (14/27).

#### *Drosophila* stocks

Flies were reared at 25C and raised on standard cornmeal/molasses food. All transgenic fly strains presented in this study including insertion sites are listed in Table 2. Transgenic fly production was performed by Bestgene, Inc., Chino Hills, CA. Other transgenic fly strains used in this study with Bloomington stock numbers in parentheses: *yw*; *QUAS-mtdTomato-3XHA* [7] (30004; 30005), *w*; *20XUAS-IVS-mCD8GFP* [25] (32194), *w*; *ppk-GAL4*, *UAS-mCD8GFP* [13] (8749), *yw*; *Rh6-GAL4* [29] (7459), *w*; *scratch-GAL4* [30], *UAS-mCD8.ChRFP* (27391; 27392; F. Schnoorer, unpublished), *UAS-n-syb-GFP* [31], and landing site stocks [32], [33].

#### Immunohistochemistry

Larva were dissected on Sylgard coated slides using minuten pins. After dissection, larvae were fixed in 4% paraformaldehyde for 30 minutes at room temperature and washed 3X two minutes in PBS. Larvae were then blocked in PBSTNGS (PBS +2% Triton X-100 +5% normal goat serum) for ≥ 1 hr at room temperature, incubated with primary antibody diluted in PBSTNGS overnight at 4C, washed 5X two minutes in PBS, incubated with secondary antibody diluted in PBSTNGS overnight at 4C, washed 5X two minutes in PBS, before imaging on a Leica TCS-SP confocal microscope. Primary antibodies used in this study were rabbit anti-GFP Abfinity mAb (Invitrogen-Cat # G10362; 1∶200), mouse anti-GFP mAb3E6 (Invitrogen-Cat # A-11120; 1∶200), Rat anti-HA mAb 3F10 (Roche-Cat # 11 867 423 001; 1∶200), and Rat anti-CD8a (Invitrogen-Cat # MCD0800; 1∶100). Secondary antibodies used in this study were donkey anti-Rabbit DyLight 488 (Jackson Immunoresearch-Cat # 711-485-152; 1∶500), goat anti-Rabbit Alexa Fluor 488 (Invitrogen-Cat # A-11034; 1∶500), goat anti-mouse Alexa Fluor 488 (Invitrogen-Cat # A-11029; 1∶500), and goat anti-rat Alexa Fluor 568 (Invitrogen-Cat # A-11077; 1∶500)

### Image Quantitation

Images were acquired with a Zeiss Lumar fluorescent stereomicroscope equipped with a QiCam camera and QCapture software. Images being compared were acquired using identical microscope and software settings. Quantification was done on iVision software. Background levels were subtracted uniformly and equally between comparison images and ratios of fluorescence intensity were obtained by dividing the summation of total pixel intensity across the ventral nerve cord.

## Supporting Information

Figure S1 **Donor vectors for Gateway MultiSite cloning.** A) The six pDONR vectors from the Gateway MultiSite Pro Plus kit. All six pDONR vectors are kanamycin resistant and are highly similar except for their distinct attP sites. The attP-containing donor vectors are used in the BP reaction with attB flanked DNA fragments (typically generated by PCR) to create entry clones. During the BP reaction the attB and attP sites are converted to attL and attR sites. attP sites of the same color indicate recombination compatibility of their corresponding attL and attR sites in the LR reaction. This same color scheme is used in Figures 1, 3, and 10. The BP reactions for all six donor vectors use the BP Clonase II enzyme mix.
(TIF)

Figure S2 **Four tandem repeats of mCherry or eGFP fused to n-syb exhibits increased fluorescence intensity as compared to fusion of one copy.** A-D) Representative confocal images of A) *yw*; *5XQUAS-n-syb-mCherry-HA*/*+*; *nompC-QF*/*+*; B) *yw*; *5XQUAS-n-syb-mCherry-HA*; *nompC-QF*/*+*; C) *yw*; *5XQUAS-n-syb-eGFP-Myc*/*+*; *nompC-QF*/*+*; D) *yw*; *5XQUAS-n-syb-4XeGFP-Myc*/*+*; *nompC-QF*/*+* age-matched third instar larva ventral nerve cords imaged via direct fluorescence. The images in A) and B) were acquired using identical confocal settings with 568 nm excitation as were the images in C) and D) with 488 nm excitation. Scale bar: 100 µm.
(TIF)

Table S1 attB primer sequences for Gateway MultiSite cloning. The attB sequences flank gene-specific sequences in the oligonucleotides used to generate the PCR products that will be used as substrates in the BP reaction. Since only specific combinations of attB and attP sites are functional in the BP reaction, the attB sites must be chosen based on compatibility with the attP sites of each pDONR vector.
(XLSX)
