# Supplementary figures and images for: A Gateway MultiSite Recombination Cloning Toolkit
Source: PLoS One. 2011 Sep 9;6(9):e24531. doi: 10.1371/journal.pone.0024531 (PMC3170369; doi:10.1371/journal.pone.0024531)

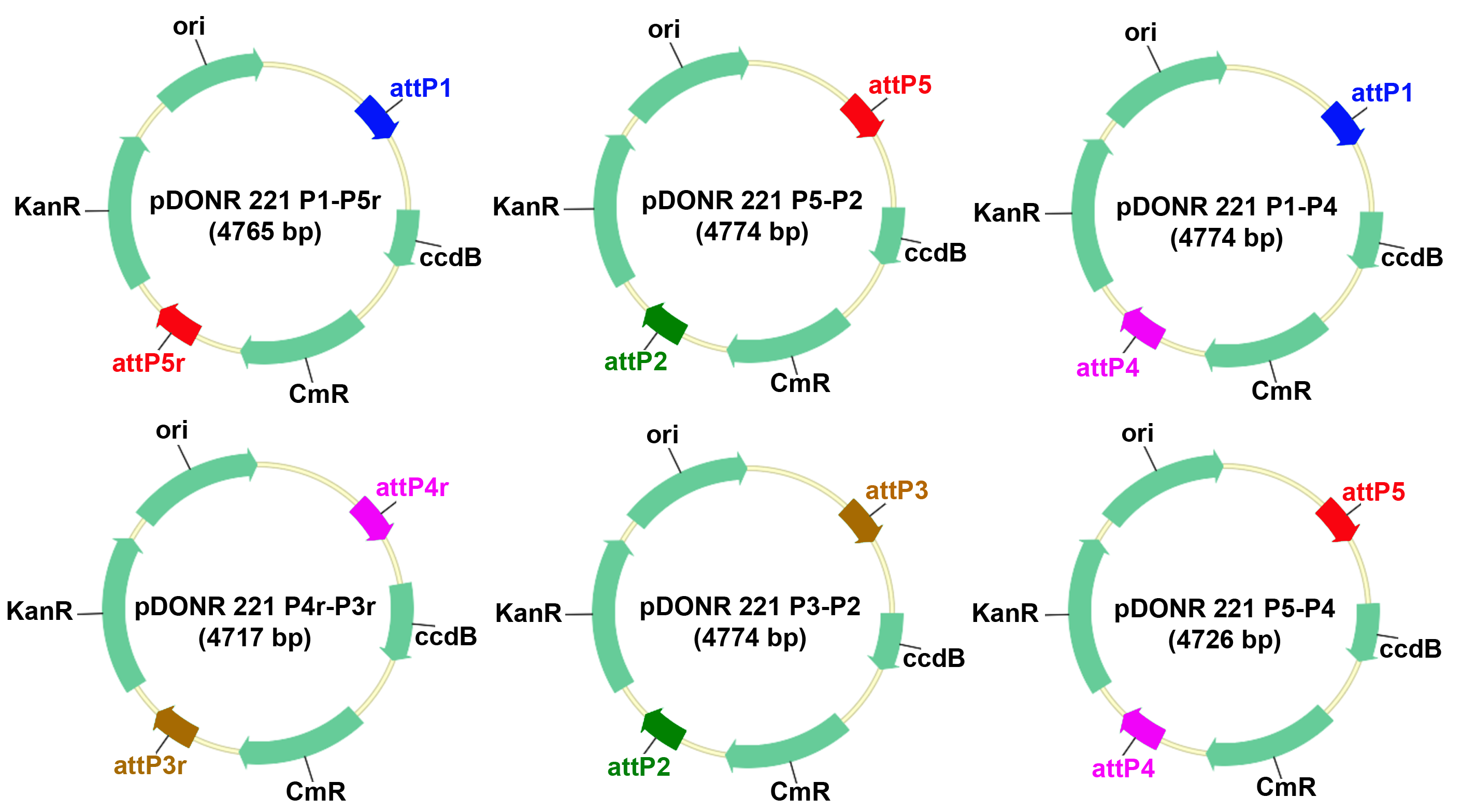

Supplement: Figure S1 — Donor vectors for Gateway MultiSite cloning. A) The six pDONR vectors from the Gateway MultiSite Pro Plus kit. All six pDONR vectors are kanamycin resistant and are highly similar except for their distinct attP sites. The attP-containing donor vectors are used in the BP reaction with attB flanked DNA fragments (typically generated by PCR) to create entry clones. During the BP reaction the attB and attP sites are converted to attL and attR sites. attP sites of the same color indicate recombination compatibility of their corresponding attL and attR sites in the LR reaction. This same color scheme is used in Figures 1, 3, and 10. The BP reactions for all six donor vectors use the BP Clonase II enzyme mix. (TIF) [file pone.0024531.s001.tif]

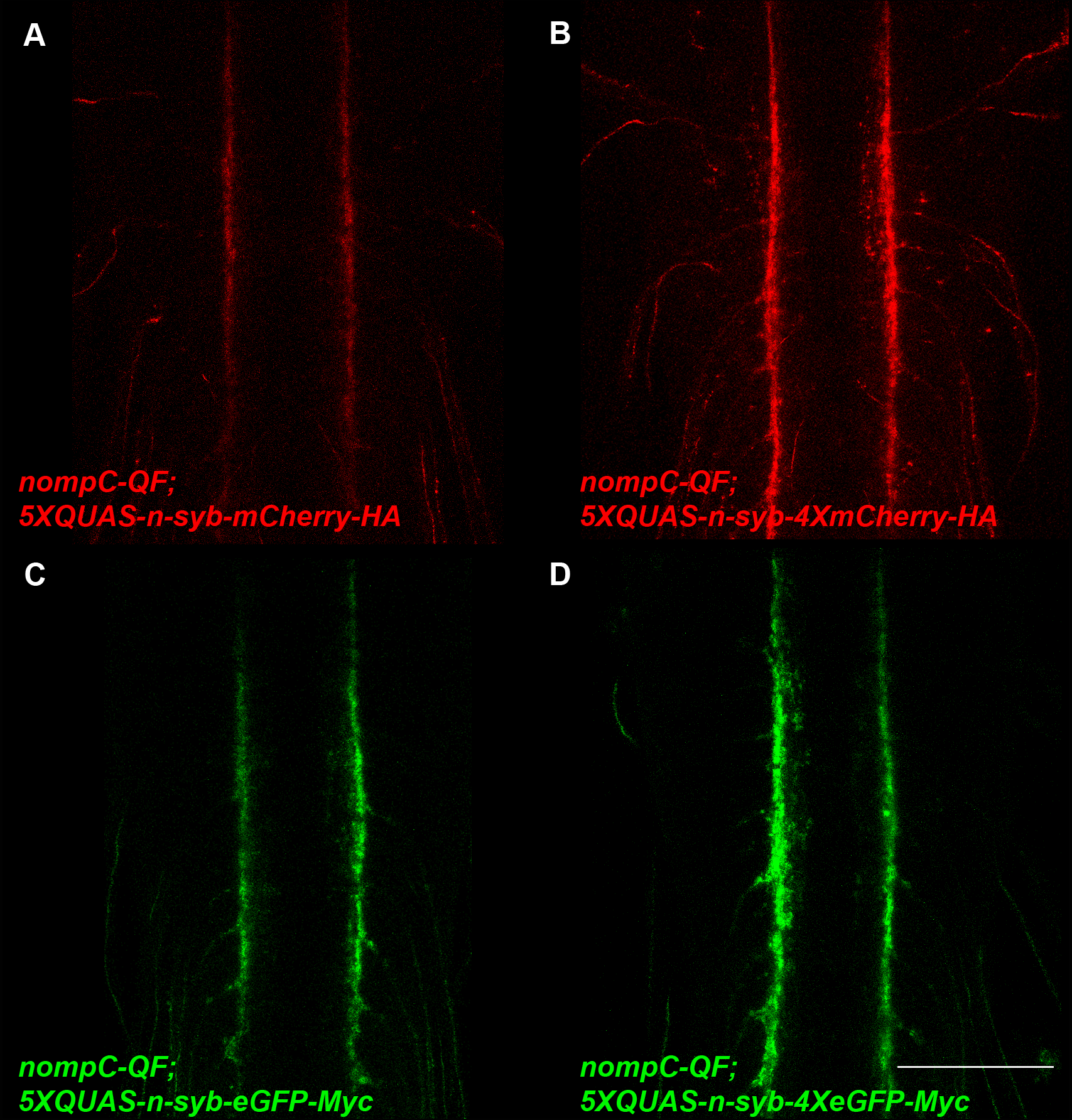

Supplement: Figure S2 — Four tandem repeats of mCherry or eGFP fused to n-syb exhibits increased fluorescence intensity as compared to fusion of one copy. A-D) Representative confocal images of A) yw; 5XQUAS-n-syb-mCherry-HA/+; nompC-QF/+; B) yw; 5XQUAS-n-syb-mCherry-HA; nompC-QF/+; C) yw; 5XQUAS-n-syb-eGFP-Myc/+; nompC-QF/+; D) yw; 5XQUAS-n-syb-4XeGFP-Myc/+; nompC-QF/+ age-matched third instar larva ventral nerve cords imaged via direct fluorescence. The images in A) and B) were acquired using identical confocal settings with 568 nm excitation as were the images in C) and D) with 488 nm excitation. Scale bar: 100 µm. (TIF) [file pone.0024531.s002.tif]
